# Post Partem Hemorrhage

**Published:** 1876-12

**Authors:** John W. Booth


### POST PARTEM HEMORRHAGE.

By JOHN W. BOOTH, M.D.

The first case of post partem hemorrhage on record terminated fatally.

It bereft a distinguished person of his favorite wife, for whom he had served her father seven years, and occurred more than three thousand years ago, near the spot where the Wise Men of the East worshipped the Saviour of mankind 1,732 years afterward.

In the 35th chapter, 16, 17 and 18 verses of Genesis, we read the inspired account of it in the following words:

(16.) "And they (Jacob's family) journeyed from Bethel, and there was but a little way to come to Ephrath: and Rachel travailed, and she hard labor." (17.) "And it came to pass, when she was in hard labor, that the midwife said unto her, Fear not, thou shalt have this son also." (18.) "And it came to pass, as her soul was departing she called his name Benoni, but his father called him Benjamin."

Rachel evidently survived the birth of the child long enough to be informed of its sex and to suggest a name, and after so hard a labor, we may naturally suppose there followed uterine inertia from exhaustion, with partial or entire separation of the placenta, the midwife being entirely ignorant of the flooding, as the blood did not flow externally, perhaps.

Modern science had not then, of course, thrown any light upon post partem hemorrhage.

The most important case, especially from a political point of view, and one that put the whole British empire in sack-cloth and ashes, occured to the Princess Charlotte, heiress apparent to the English crown.

We are told that the Accoucheur, because of her high rank, was too modest, falsely so-called, to put his hand on her abdomen and grasp the uterus, to ascertain if it was contracted, and by his false delicacy sacrificed the nation's favorite.

Dr. Gooch, in his book on midwifery, says: Nature provides against the danger of hemorrhage by a process at once simple and effectual. The child being born, the uterus, which wa before an immense sack filling the belly, contracts into a hard ball, situated just above the pubes. * * * * By this contraction the mouths of the vessels are closed in that part of the internal surface of the uterus to which the placenta had previously been attached; thus, the danger of hemorrhage, on the separation of the placenta, is averted by that contraction of the uterus by which its separation is produced.

If nature always accomplished her design in this manner, and the uterus kept up the same contraction, we should never be called upon to encounter this form of flooding.

Sometimes there is flooding before the placenta is delivered; but this cannot happen unless the placenta is detached at some point, and this detachment is always caused by uterine action—that is to say, by an ineffectual effort to drive it off, unless it may have been caused by some mechanical injury.

When there is flooding before the extrusion of the placenta, one of the following conditions must exist: There may be partial separation while the uterus may posess little or no contractile power, or a greater tonic power, or while a part of the placenta is separated a part may be preternaturally adherent, and there may be full contractile power, or the uterus may act but feebly. There may be entire or partial separation while there is complete exhaustion. Last, the neck may enjoy its full tonic power while the body and fundus are in a state of complete inertia.

In all these conditions, but one, we may hope to induce sufficient contractions to expel the placenta, and close the sinuses, by external manipulation of the uterus. If this is not sufficient the hand may be cautiously introduced, or perhaps the application of cold, etc., may answer. But when a part or the whole of the mass is morbidly adherent, the hand must be introduced, and it must be dealt with in a manner, the details of which I need not enter into here. In like manner in the irregular contractions above noticed, hour-glass contraction, the hand must be introduced to overcome the contraction of the circular fibres, and stimulate the longitudinal ones to contract.

Systematic writers on midwifery caution us against the withdrawal of the hand before contraction is distinctly perceived, and urge that even then it be withdrawn very gradually, and only as it is followed up and pressed on by the uterus with the placenta.

This direction has been handed down from one generation of obstetricians to another, and I have always held it sacred. Dewees says: "If the uterus regain its wonted power, the hand with the placental mass will be expelled almost immediately from its cavity; but even when this effect is perceived the hand should not be permitted to leave it too suddenly."

I once, however, verified by accident that the hand may be withdrawn very suddenly and unceremoniously without serious consequences. In 1859 I delivered a very powerful, athletic woman, a farmer's wife, noted for the unusually large size of her children at birth, of a still-born male child weighing fifteen pounds. The labor was exceedingly hard, and after the head had passed it was with great difficulty that I could deliver the shoulders. They were eight inches across. (Measured by A. H. Cook, now Register of Deeds of this county.)

This labor was soon followed by some flooding and retained placenta, and I found that the mass was imprisoned in the upper part of the uterus by hour-glass contraction. Introducing my hand very timidly and cautiously, for it was the first time that I had undertaken that operation, I had overcome the stricture, and was passing my hand around the placenta, when she, as if frantic with pain, screamed at the top of her voice, and seizing me by the beard, gave a hard and sudden jerk. Taken by surprise and thrown entirely off my guard, by the physical pain, my beard being exceedingly tender, I withdrew my hand as suddenly and quickly as I could, without a thought of what might follow. I was very much alarmed when I thought of what I had done, but the organ contracted immediately and firmly, and the placenta was found entirely expelled from the genitalia. This woman did well and died several years afterward of diabetes melitus, having shortly previous to her death lost the sight of both of her eyes from cataract.

If from deficient action of the uterus or some fault of the circulation or of the blood, there should be flooding after the delivery of the placenta, there arises a somewhat new state of things, to be met usually in a manner similar to the treatment of the same accident happening before its delivery.

As before, the first and very frequently the only means necessary is to grasp and rub or knead the uterus externally through the abdominal parietes. This process is now never overlooked, and we are surprised when first informed that it is not mentioned till 1792, when Mons. Dape, a Parisian Accoucheur, first recommended it in the following words:

"Il ne faut qui portes les deux mains sur la region hypogastrique, et comprimer mollement le corps de la matrice par un movement tautot circulaire tautot de droit a gauche, de gauche a droiter, differeus mouve nuns sont absolument. Tousce a cause des differens plans de fibres que 'sentrecroisent et forment une espice de reseau."

Obstetricians during the whole of the present century, I believe, have used only one hand for this purpose, it being more convenient. In a vast majority of cases, this is sufficient to cause contraction and bring safety. If it fail, ergot may be given, but that is thought to do better when taken just before the passage of the head. Then the hand may be introduced, and if the part where the placenta was attached can be found, it may be pressed between the fist within, and the hand externally, then cold internally and externally applied, so as to produce a shock, &c., or the order may be reversed. Notwithstanding all these weapons tried and true, the flooding in exceptional cases goes on unabated, and that brings us to intra uterine injections and solids introduced with the hand to arrest flooding, such as vinegar and water, lemon juice, perchloride of iron, tincture iodine, etc.

For a few years past the perchloride of iron in the treatment of certain cases of Post Partem Hemorrhage, has been exciting a good deal of interest in the profession everywhere, but especially in Great Britain.

It seems, according to a Norwegian professor, F. C. Fay, M. D., of the University of Christiana, the late Professor d'Outrepout of Wurzburg, is entitled to the credit of first having used and written about it, upwards of thirty years ago. Dr. Fay says:

"Since that time and during the whole of the time, I have been appointed to our clininal Lying-in-Hospital. I have frequently used the method, and usually with decided benefit. My printed reports from our maternity as far back as 1847, and the following years, bear testimony of the beneficial effect of the fore-named injection."

To Dr. Robert Barnes, of London, however, rightly belongs the honor of having first brought it prominently before the professional puolic. The lectures in which it was taught were delivered, I suppose, some ten or twelve years ago, but did not excite so much discussion till later. I have not the early literature of the subject, but in the *British Medical Journal* of January 11, 1873, Dr. B. says:

"The great lesson to learn is to take courage and use the styptic in time, that is before the vital power has sunk too low. It was not expected that a remedy powerful enough to save life under the last extremity, should be entirely free from danger. But I have seen so many women bleed to death, and have seen so many saved by the timely use of the iron injection, that I am much more afraid of the bleeding than the remedy. * * * If occasionally death follows, and is apparently accelerated by the iron injection, we have on the other hand to remember that it was used when the patient was likely to die, even if nothing were done, and that even under these unpromising conditions *many lives, to all appearances doomed, have been saved.*"

Speaking of its danger, he says very pertinently, harmless remedies fail, as a rule, in great emergencies. But cold water injection fail not because of its harmlessness, for the shock and impression which it causes are extremely dangerous, but it fails because nervous power being exhausted, it cannot excite uterine contraction, and has no other virtue in arresting hemorrhage.

The styptic seizes the blood at the mouths of the vessels, constringes the inner surface of the uterus, seals up the mouths of the vessels with coagula and closes them, giving the system opportunity to rally, and the contractile power of the uterus opportunity by and by to return.

Since the publication of Dr. Barnes' teaching on this subject, there has been a somewhat warm and protracted discussion growing out of the doctrine set forth by him, and the contestants, although standing at the very head of the profession, have sometimes been betrayed by their zeal into personalities, and have sometimes been more emotional in their rhetoric than is justifiable in discussing strictly scientific questions. It is claimed by perchloride of iron supporters that it almost never fails to arrest the hemorrhage, after all known means have failed, and to cause the womb to contract permanently. It is said by its advocates to be specially applicable to patients that have previously been in bad health and are anæmic, that is when the fibrin is deficient in the blood.

Dr. Lambe Athill, Vice President of the Obstetrical Society of Dublin, in an account of five cases that occurred in his private practice, in which perchloride was used, says that he had an opportunity of judging, not only of the previous state of health, but of tracing the subsequent history of each patient. "In all it may be fairly assumed the blood was in an abnormal condition, probably destitute of its proper proportion of fibrin. In three of the four patients (one was treated twice) pregnancy subsequently ensued, which fact, he claims, clearly shows that the uterus was in no way injured. In four of the five cases, notwithstanding the great loss of blood and previous bad health, there was no unfavorable symptom. In one, death followed. In that case he makes it probable the patient would have died, whether the perchloride had been used or not."

He advocates its use after the ordinary means have failed, and considers those means the same that are usually taught us.

Dr. Hill Ringland uses the solid perchloride, applied to the bleeding surface with the hand, because he thinks the danger of injecting the fluid into the sinuses of the uterus, or into the cavity of the abdomen by the fallopian tubes is thereby obviated. Some other observers corroborate his experience.

Dr. Moore Madden, after an account of ten cases treated with the iron injection, of whom eight recovered and two died, concludes that these cases are on the whole in favor of the employment of the remedy, in some cases of post partem hemorrage, which resist all other treatment.

The testimony of many others, of pretty nearly equal eminence, is nearly about the same.

Dr. Snow Beck, on the other hand, and his adherents, not only deny that its direct styptic effect seals up the bleeding uterine vessels, or that it stimulates the uterus to permanent contraction, and assert and prove to their own satisfaction that there is great danger by forcing the injected fluid into the patulous uterine sinuses, that embolism will be produced or peritonitis, by its entering the abdominal cavity by the fallopian tubes.

I cannot rest any opinion of this agent on my own experience, and might well close what I have to say on it by a quotation or two from Dr. Jouline Gazette, as cited in the *British Obstetrical Journal*. He says:

"I acknowledge that I am not in favor of them. (Perchloride injections.) *Apropos* of this, I said, when it is a question of life and death, we ought to be dispassionate and free from selfish thoughts. Under the pretext of progress, let us not introduce scientific novelties, which all the world acknowledge are dangerous, but which few are prepared to certify as useful.' He quotes from the *Obstetrical Journal* that the opinion of those that have tried it is almost unanimously favorable, and that those who doubt its efficacy or think it dangerous, are represented by Dr. Beck in England and himself in France. He adds, that sufficient fatal cases were brought forward in the discussion of the Obstetrical Society to justify his fear, and prove that the agent is a dangerous one. From the manner in which the facts were dwelt on, I was convinced, he continues, that they were stepping on new and imperfectly known ground. An absence of rules and method are perceived, and a want of precision in the knowledge of indications to be fulfilled. It is truly said that this agent ought only to be employed as a last resource, but on observation, an impatience in making experiments is noticed, which leads to a neglect in using well known means which should have been tried at first. His conclusion is, that he does not allow his mind to be made up, and that he will praise inter-uterine injections of perchloride of iron soon enough when the English have obtained more unequivocal results.

After examining all the authorities on the subject, I should doubtless give the perchloride a full trial if I had a case in which all other means at my command had failed, and the patient was still flooding, and should at the same time feel that I was using a dangerous weapon, the use of which required the exercise of much discretion. There is a form of post partem hemorrhage mentioned by authors of half a century ago, that I ought to mention, where the uterus is pretty firmly contracted after the delivery of the placenta in plethoric women, whose circulatory system is very much excited. Blood-letting and arterial sedatives are recommended. I do not recollect to have met with a case. I have only met two cases that did not yield to the usual treatment. They were arrested by tinct. iodine. In 1859 I attended a married woman about twenty years old, in her first labor. She enjoyed as good health as is customary during her pregnancy, except a pretty tight attack of nephritic colic a few weeks previous to her confinement. After she had been in labor a day or two, and when the head had been resting on the perineum for several hours without any perceptible advancement on account of regidity and irregular contractions, I sent for the late Dr. Russell, of this county. He gave her some ergot without any effect, then delivered her instrumentally of a dead male child rather above the average size. There was a smart dash of hemorrhage immediately from inertia and partial detachment of the placenta. Friction caused contraction, and the mass was removed, but very soon the uterus relaxed with a gush. Friction contracted it again and again, but I could feel it soften and relax under my hand, assuming very different forms at different times. Various remedies were tried, including the injection of cold water. The flooding continued in spite of all, till the flickering spark of life seemed extinct. The doctor suggested tincture of iodine, on account of its known efficacy in hemorrhage of the bowels. We injected it into the organ, and the uterus contracted immediately and permanently, and the patient reacted slowly and perfectly. She rallied gradually, and recovered without any bad symptom except that her bladder had to be emptied for several days with the catheter. She is now a widow with four children.

The other case was a short, stout, muscular woman, the mother of eight or ten children. During her pregnancy, however, and for a while previously, she had been very poorly; but all that I know about it is that she had an initiating discharge from the vagina. She fell in labor August 9th, 1871, rather amæmic, or hydræmic, with a good deal of serous effusion in the cellular tissue. During the first stage the pains were irregular, and dilatation was also irregular, about half of the circumference of the os being as thick as my hand, while the other was not remarkably thick. The placenta was found at the top of the vagina, and was taken away as usual, the uterine globe being tolerably firm. My attention was directed in twenty minutes to the severity and frequency of the after pains. I removed the clots, expecting that all would then be right, but about that time the uterus relaxed and flooding began. After going over all the usual means, I had recourse to the tinct. iodine injection with the happiest result. This patient had a sort of subacute or chronic hysteritis afterwards. Her uterus was evidently diseased before.

I am not aware that this treatment was ever recommended in post partem hemorrhage before I reported these cases some years ago in one of the medical journals. I have forgotten which, and cannot find it. And previous to the first case I do not know that it had been used in uterine hemorrhage of any kind. I have frequently used it on the advice of authors with the happiest result in menorrhagia and metrorhagia, both by injection and painted on the inside of uterus through the speculum, or passed through a canula on a brush.

These two cases are the only ones in which I know of the tinct. iodine having been used in post partem hemorrhage, and all that I claim is that, if it acts in other cases as it did in these two, it is preferable to the astringent styptics because it does not produce any coagula, wnich are subject to place the patient's life in jeopardy from septicermia after the danger of flooding is past, as shown by the following case of Dr. Playfair: A few days after the debate on the merits of the perchloride, in the Obstetrical Society of London, Dr. P. says he firmly believes he saved the life of a patient by it, (the perchloride) yet grave and alarming symptoms followed its use. When the iron was injected, although the hand was in the uterus, and clots within it had been as much as possible removed, blood was pouring out abundantly. The powerful astringent at once corrugated all the blood and coagula it came in contact with, and these hardened clots filled up the uterus and the canal of the vagina. In due course, these began to decompose, and septic absorption took place, etc. This patient's life was saved by the Doctor gradually breaking down and removing the clots.

Iodine does not coagulate fibrin, and therefore could not be followed by this accident. I believe some American Obstetricians prefer the persulphate to the perchloride.

